# Parental Renovascular Hypertension-Induced Autonomic Dysfunction in Male Offspring Is Improved by Prenatal or Postnatal Treatment With Hydrogen Sulfide

**DOI:** 10.3389/fphys.2019.01184

**Published:** 2019-09-19

**Authors:** Qi Guo, Xiaohong Feng, Hongmei Xue, Sheng Jin, Xu Teng, Xiaocui Duan, Lin Xiao, Yuming Wu

**Affiliations:** ^1^Department of Physiology, Hebei Medical University, Shijiazhuang, China; ^2^Department of Laboratory Diagnostics, Hebei Medical University, Shijiazhuang, China; ^3^Hebei Key Laboratory of Animal Science, Hebei Medical University, Shijiazhuang, China; ^4^Hebei Collaborative Innovation Center for Cardio-Cerebrovascular Disease, Shijiazhuang, China; ^5^Key Laboratory of Vascular Medicine of Hebei Province, Shijiazhuang, China

**Keywords:** hypertension, autonomic dysfunction, arterial baroreflex sensitivity, fetal programming, angiotensin II

## Abstract

Increasing evidence indicates there is a strong association between parental health during pregnancy and incidence of cardiovascular disease in adult offspring. Recently, hydrogen sulfide (H_2_S) has been demonstrated to be a powerful vasodilator of the placental vasculature, improving intrauterine growth restriction. In this study, we investigated whether parental hypertension induces autonomic dysfunction in male adult offspring, and the H_2_S mechanism underlying this autonomic dysfunction. 2-kidney-1-clip method was employed to induce parental hypertension during pregnancy and lactation in rats. Basal blood pressure (BP) and autonomic function of male offspring in adulthood was evaluated. Additionally, either maternal hypertensive dams or their male offspring after weaning were treated with H_2_S to determine improving effects of H_2_S on autonomic dysfunction. The BP was significantly increased in male offspring of renovascular hypertensive dams when compared to that in offspring of normotensive dams. The offspring of renovascular hypertensive dams also exhibited blunted baroreflex sensitivity, increased sympathetic effect and sympathetic tonus. Western blotting analysis revealed downregulation of endogenous H_2_S catalyzed enzyme and upregulation of angiotensin Ang II type 1 receptor (AT1R) pathway in the nucleus tractus solitarius and rostral ventrolateral medulla, two hindbrain nuclei involved in BP and autonomic regulation, in these offspring. Either prenatal or postnatal treatment with H_2_S improved the adverse effects. The results suggest that parental hypertension results in elevated BP and autonomic dysfunction in adult male offspring through activation of AT1R pathway and inhibition of endogenous H_2_S production in the brain.

## Introduction

It is established that chronic hypertension during pregnancy leads to adverse consequences to both mothers and fetuses (Lykke et al., 2009). Parents tend to have children later in life when the incidence of obesity, and renal disease associated hypertension are increased. Many studies have shown that an adverse intrauterine environment, mothers with pre-eclampsia or chronic hypertension, inflammation, undernutrition, reductions in sleep during pregnancy, diabetes, and so on, results in an increased risk of cardiovascular diseases in the adult offspring (Barker, 1990; Lazdam et al., 2010; Staley et al., 2015; Wu et al., 2015). These studies also indicate that sympathetic overactivation, autonomic dysfunction and inflammation may contribute to the development of hypertension in the offspring of mothers with a variety of insults during pregnancy and/or lactation.

Several studies have revealed that renin-angiotensin-aldosterone system (RAAS) participates in mediating the fetal programming of hypertension (Xue et al., 2017). A maternal low protein diet during pregnancy led to upregulated expression of the angiotensin II receptor 1 (AT1) in the adrenal gland (Bogdarina et al., 2007). Maternal high-salt diet during pregnancy resulted in angiotensin-related cardiac changes in offspring (Ding et al., 2010). In contrast, low protein diet mothers treated with an angiotensin-converting enzyme inhibitor or an angiotensin (Ang) II receptor blocker reversed elevated blood pressure (BP) in the offspring (Mansuri et al., 2015). Studies demonstrate that the early life NF-κB dyshomeostasis induced by prenatal inflammatory exposure plays an essential role in the development of EH through triggering RAS over-activity (Deng et al., 2016). Therefore, RAAS combined with inflammation attributed to fetal programmed hypertension. One of the primary mechanisms involved in renovascular hypertension induced by two-kidney-one-clip (2K1C), is the activation of renin-angiotensin system (RAS). In the present study, renovascular hypertension was employed to induce secondary hypertension in parental rats.

It has been shown that arterial baroreflex (ARB) contributes to stability of BP, and that autonomic dysfunction is involved in the development of hypertension (La Rovere et al., 2013; Ryder et al., 2016). Nucleus tractus solitarius (NTS) and rostral ventrolateral medulla (RVLM) are two important nuclei involved in regulation of cardiovascular function and play important roles in controlling of BP and autonomic function. Whether activation of the RAAS in the NTS and RVLM exacerbates autonomic function to facilitate the development of hypertension in offspring from dams with renovascular hypertension is not clear (Averill and Diz, 2000; Baltatu et al., 2001).

Hydrogen sulfide (H_2_S) is a newly labeled endogenous gaseous signal molecule, similar to nitric oxide and carbon monoxide. Cystathionine beta synthase (CBS) and cystathionine gamma lyase (CSE) enzymes catalyzed cysteine residues to produce endogenous H_2_S. The enzymes have tissue-specific distribution and the central nervous system mainly express CBS (Hu et al., 2011). Previous studies found hydrogen sulfide improves baroreceptor reflex (Guo et al., 2016), inhibits sympathetic outflow (Guo et al., 2011), relaxes thoracic aorta and renal arteries (Xue et al., 2015), and improves cardiac function of hypertensive rats (Liu et al., 2016). Moreover, others have demonstrated endogenous H_2_S is essential for maintaining placental function. Decreasing activity of CSE/H_2_S pathway could cause pre-eclampsia and fetal growth restriction in pregnant women (Wang et al., 2014). In the kidney, we also found prenatal or postnatal administration with H_2_S increases methylation of AT1b gene, down regulating protein of AT1R in offspring of parental secondary hypertension (Guo et al., 2017). However, little investigation has occurred pertaining to the central effect of H_2_S prenatal intervention on fetal-programmed hypertension.

Our hypothesis is the prenatal secondary hypertension induces autonomic dysfunction in offspring and the related central mechanisms are through activation of AT1R pathway and inhibition of endogenous H_2_S production in the brain of the male offspring.

## Materials and Methods

### Animals

All animals were housed in 12 h light/dark cycle, at standard room temperature of 25 ± 3°C. Food and water were freely available. All protocols and procedures used in this study were reviewed and approved by the Animal Management Rule of the Ministry of Health, People’s Republic of China (documentation number 55, 2001), the Animal Care Committee of Hebei Medical University and in accordance with NIH guidelines (Guide for the care and use of laboratory animals) (NIH Publication No. 85-23, revised 2011).

### Parents and Hypertension Model

We selected 7 weeks old male and female Sprague-Dawley (SD) rats, weighting 160–180 g, to prepare renovascular hypertension (RVH). Rats were anesthetized using pentobarbital sodium (50 mg/kg). The absence of a withdrawal response to a paw pinch was used to confirm adequate anesthesia. The left kidney was exposed via laparotomy and the left renal artery was carefully separated from the left renal vein and connected tissues. Then the left renal artery was clipped by a rigid U-shaped solid clip. The stainless steel clip with an opening width of 0.25 mm (Aowang Medical Instrument Company, Shanghai, China), resulting in partial occlusion of renal perfusion. Thereafter, animals were administered with intramuscular procaine penicillin G (30,000 U/100 g) and meloxicam (1 mg/kg) for 3 days. Systolic blood pressure (SBP) was measured each week for 5 weeks after surgery by tail-cuff plethysmography (Chengdu Instrument Factory, Sichuan, China) in conscious rats. The rats were trained to become accustomed to the procedures for blood pressure measurement before the experiments. Each rat was placed on a heating pad for 15–20 min to promote vasodilatation of the tail artery prior to measurement of SBP.

### Study Design

**FIGURE 1:**
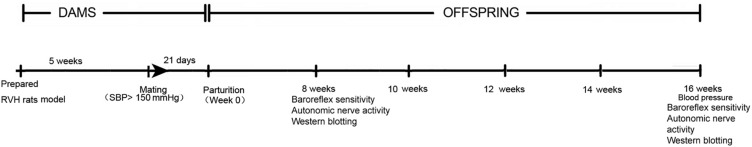
A timer shaft of the protocol in the experiment.

Ten pairs Sprague-Dawley were used for mating, and thirty pairs of RVH rats were chose to mate. Each female rat was separately mated overnight. Day of pregnancy was defined as the day when spermatozoa were found in a vaginal smear. In the experiment, there were four groups, every group has ten pairs of parents: The offspring of SD rats was Control group (*n* = 10 l). F_1N_F_2N_ meant the offspring of RVH rats (*n* = 10 l). F_1H_F_2N_ meant maternal RVH rats treated with NaHS (H_2_S donor, 56 μmol/kg per day, intraperitoneal (i.p.) injections) during pregnancy and lactation periods and the offspring without NaHS (*n* = 10 l). On first day of pregnancy, each mother was received NaHS treatment. F_1N_F_2H_ group meant maternal RVH rats were treated without NaHS and the offspring with NaHS (H_2_S donor, 56 μmol/kg per day, intraperitoneal (i.p.) injections) after weaning at 4 weeks (*n* = 10 l) (“1” represents first generation, “2” represents the second generation, “N” represents normal saline, and “H” represents hydrogen sulfide). Each experimental group was composed of individual subjects that were randomly selected from different litters. In addition, the protocol of the experiment was showed in a timer shaft (Figure 1).

### Offspring

#### Blood Pressure Measurement in Conscious Offspring

Systolic blood pressure (SBP), diastolic blood pressure (DBP) and mean artery pressure (MAP) were measured in conscious offspring. Pentobarbital sodium (50 mg/kg) was used for anesthesia. Polyethylene catheters (5 cm PE10 combined with 25 cm PE 50, SCI, United States) filled with sterile saline containing heparin (50 U/ml) and penicillin G (2000 U/ml) were inserted into the femoral artery and vein, and its tip was placed in the abdominal aorta. The catheters were tunneled subcutaneously into the back of the neck and sealed with a plastic cap (Abdala et al., 2012; Moreira et al., 2018). All animals received a subcutaneously injection of penicillin G (30,000 U/100 g) and meloxicam (1 mg/kg). Catheters were flushed the following day with the same solution containing heparin and penicillin. 2 days after surgery, SBP, DBP and MAP were recorded by Power lab 15T, data-acquisition software, using transducer. On the day of the experiments, the catheter was connected to a pressure transducer (MLT1199; AD Instruments) coupled to a preamplifier (Bridge Amp, ML301; AD Instruments) that was connected to a Powerlab computer data acquisition system. Arterial pressure measurements were taken between 09:00 and 12:00 h by an experimenter that was blind to the animal condition. Data was acquired in conscious animals for 60 min during quiet resting periods (*n* = 12).

#### Measurement of Ang II Concentration

Radioimmunoassay was used to test Ang II concentration. Collected blood sample, then EDTA and enzyme inhibitors were added and mixed in a tube on ice. The mixture was centrifuged at 3,000 rpm for 15 min at 4°C. The plasma was collected and examined with an Ang II radioimmunoassay kit (China Institute of Atomic Energy, Beijing, China). All samples were analyzed in duplicate as the manufacturer’s instructions.

#### Baroreceptor Reflex Sensitivity in Conscious Rats

Traditional pharmacological approaches were used to assess baroreflex sensitivity in conscious animals. In order to test the baroreflex control of heart rate (HR) in conscious rats, we need to record the blood pressure and the method same as the above. HR was derived from the blood pressure waveform. Arterial pressure was increased with graded infusions of either phenylephrine (PHE; 1.0 mg/mL, Sigma, United States), Ang II (100 μg/mL, Sigma, United States) and decreased with sodium nitroprusside (SNP 1.0 mg/mL, Sigma, United States), as previously described (Xue et al., 2003). Infusion rate (3 μL/min) was monitored such that blood pressure was increased or decreased 40–50 mmHg over 30 s period. Peak values of MAP and HR in response to Ang II, PHE and SNP injections were fitted to a sigmoidal logistic equation to determine the slop, the maximum reflex tachycardia and reflex bradycardia. Data were analyzed by linear regression using Prism 6 (GraphPad, United States) and the slope of linear regression provided baroreflex gain for each animal. After data collection, the offspring were euthanized with carbon dioxide inhalation.

#### Investigation Autonomic Nervous System Function

The method of recording BP and HR of the conscious animals is the same as above. For two consecutive days we used cholinergic receptor blocker (sulfate-atropine, 2.5 mg/kg, Sigma, United States) and β1-blocker (propranolol, 4 mg/kg, Sigma, United States), the peripheral inhibition of the sympathetic and parasympathetic activities were performed in a reverse order. On the first day, resting heart rate was recorded for 30 min, and then atropine was given to the offspring. The maximum value of heart rate changes was recorded after 10–15 min. The difference between the maximum and the basal heart rate was the vagal effect. Subsequently, propranolol was injected and the new HR level was obtained after 10–15 min, to determine the intrinsic HR (IHR). On the second day, the blockade sequence was inversely induced. Propranolol was administered intravenously and the minimal HR was measured after 10–15 min, to determine the sympathetic effect. Then the HR, after 10–15 min following injection atropine, was regarded as IHR. The final IHR was obtained from the average of the 2 days. The vagal tonus was calculated as the difference between the IHR and the minimal HR obtained after propranolol injection alone. The sympathetic tonus was determined as the difference between the maximum HR obtained after methyl-atropine injection alone and IHR, as described previously (Caligiorne et al., 2008).

#### Western Blot Detection of Protein

The offspring were sacrificed by carbon dioxide inhalation at 8 and 16 weeks old. The brain was rapidly removed and put in oxygenated ice-cold artificial cerebrospinal fluid. A brain block containing the RVLM and NTS was cut and fixed on a vibrating microtome (Leica VT 1200S). Coronal slices through the NTS and RVLM were cut at a thickness of 250 μm in a freezing artificial cerebrospinal fluid. NTS and RVLM were separated in accord with Paxinos and Watson’s coordinates. Both sides of the RVLM (coordinates: 12.0-12.5 mm caudal to bregma, and 0.3–0.9 mm from the ventral surface, 2.0–2.5 mm lateral to the midline) and NTS (13.6–14.5 mm caudal to bregma, 7.4–7.9 mm below the skull surface of bregma, 0–1.5 mm lateral to midline) were collected using a stainless steel micro-punch (1 mm internal diameter) and then put them in liquid nitrogen and stored at −80°C for further analysis. The tissue was homogenized in ice-cold 100 μL lysing buffer (contained: 1 μg/mL leupeptin, 5 μg/mL aprotinin, 100 μg/mL PMSF, 1 mmol/L sodium orthovanadate, 1 mmol/L EDTA, 1 mmol/L EGTA, 1 mmol/L sodium fluoride, and 2 μg/mL β-glycerolphosphate) and then centrifuged at 15,000 × *g* for 20 min at 4°C. We collected supernatant for protein assay. Bradford assay (Generay Biotechnology, Shanghai, China) was performed to determine the concentration of protein in tissue. The protein sample was denatured at 99°C for 10 min. 15 μg protein samples were loaded in each lane. After electrophoresis, the protein was separated then transferred onto polyvinylidene fluoride (PVDF) membranes at 200 mA for 90 min at 4°C. The PVDF membrane was blocked with 0.1% Tween-20-Tris-buffered saline (TBST) containing 5% non-fat milk for 1 h at room temperature. The PVDF membranes were then incubated overnight at 4°C with anti-AT1R (1:800, Abcam, MA), anti-NOX4 (1:1000, Abcam, MA), anti-NLRP3 (1:1000, Novus, United States), anti- IL-1β (1:500, Novus, United States), anti- CBS (1:800, Santa, United States), followed by appropriate secondary horseradish peroxidase-conjugated antibody (1:2000, Proteintech Biotechnology). GAPDH (1:3000, Proteintech Biotechnology) was used as a loading control. All washes were used in TBST. Western blotting reagents (Millipore Corporation, United States) were used to detect the signal. The protein level on the nitrocellulose membrane was detected by a chemiluminescent substrate system (Sagecreation, Beijing, China). Densitometric analysis was conducted on the protein bands for quantitative comparison.

#### Quantitative Real-Time PCR

Total RNA of the NTS and RVLM were isolated using the RNeasy MiniKit (Qiagen; Germany). 1 μg RNA was used for reverse transcription with a RevertAid RT Reverse Transcription Kit (Thermo Fisher Scientific). The cDNA samples were diluted and studied at several concentrations. Two microlitres of diluted cDNA was added to a 20 μl reaction volume with SYBR Green Real-Time PCR Mix (Thermo Fisher Scientific). Real-time PCR was performed using a QuantStudio 6&7 Real-Time PCR System (Thermo Fisher Scientific). The thermal cycling conditions were as follows: 1 cycle at 95°C for 5 min, 35 cycles at 95°C for 15 s, and 60°C for 45 s. Experimental *C*_t_ values were calculated compared with a reference sample. Each sample was run and analyzed in triplicate. Primers were designed by Sangon Biotech (Shanghai, China). The sequences of the primers are listed in Table 1. GAPDH was used as an internal standard to normalize the expression level of each mRNA.

**TABLE 1:** Sequence of primers used in RNA quantitation experiments.

**AT1**	5′ (F) CACAAC CCT CCC AGA AAG TGA TC 5′ (R) GAT GAT GCT GTA GAG GGT AGG G
**GAPDH**	5′ (F) CAT CCC AGA GCTGAA CGG GAA G 5′ (R) GTC CTC AGTGTA GCC CAG GATGC

### Data Analysis

All data were expressed as means ± SD. One way ANOVA analysis of variance followed by Student-Newman-Keuls test and Dunnett’s *t*-test were used when multiple comparisons were performed. To analyze baroreflex responses, the absolute value of heart rate for each dose of Ang II, PHE, or SNP was plotted against the corresponding value of MAP for each rat, and the data were subjected to linear regression analysis. Individual subjects in each experimental group were chose from different litters randomly. The level of significance was set at *P* < 0.05.

## Results

### The BP in Conscious Male Offspring

**FIGURE 2:**
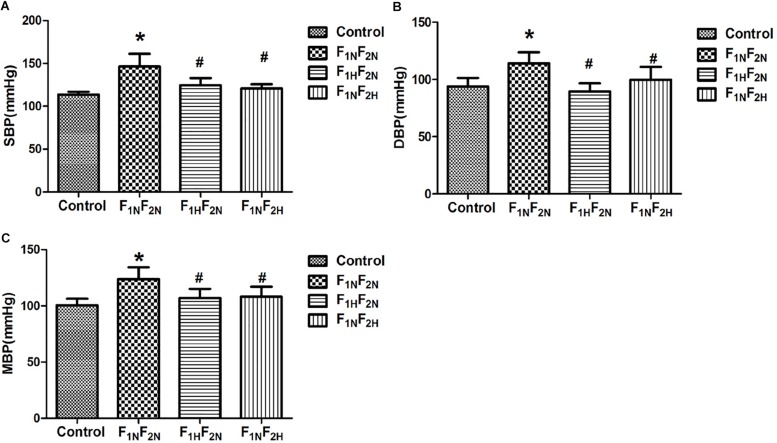
Systolic blood pressure (SBP, in mmHg) diastolic blood pressure (DBP, in mmHg) and mean arterial blood pressure (MAP, in mmHg) in conscious male offspring at 16 weeks from Control, F_1N_F_2N_ (the offspring of RVH rats), F_1H_F_2N_ (RVH rats treated with NaHS, offspring without NaHS), and F_1N_F_2H_ (maternal RVH rats treated without NaHS and the offspring with NaHS after weaning). **(A)** SBP, **(B)** DBP, **(C)** MAP. ^∗^*P* < 0.05 compared with Control group. ^#^*P* < 0.05 compared with F_1N_F_2N_ group (*n* = 6–8). Values are means ± SD.

The SBP, DBP and MAP were measured at 16 weeks in male offspring Compared with control group at 16 weeks, SBP, DBP and MAP in offspring of renovascular hypertensive rats were significantly increased from 113.59 ± 3.29 mmHg to 146.34 ± 10.97 mmHg, 93.82 ± 7.53 vs. 112.51 ± 8.68 mmHg, 100.41 ± 5.8 vs. 123.78 ± 8.5 mmHg *P* < 0.05. However, maternal exogenous administration with H_2_S during pregnancy resulted in lower BP level in the offspring. Likewise, in the offspring treated with H_2_S after weaning, the BP was reduced to normal at 16 weeks (Figures 2A–C).

### Baroreflex Sensitivity in Male Conscious Offspring

**FIGURE 3:**
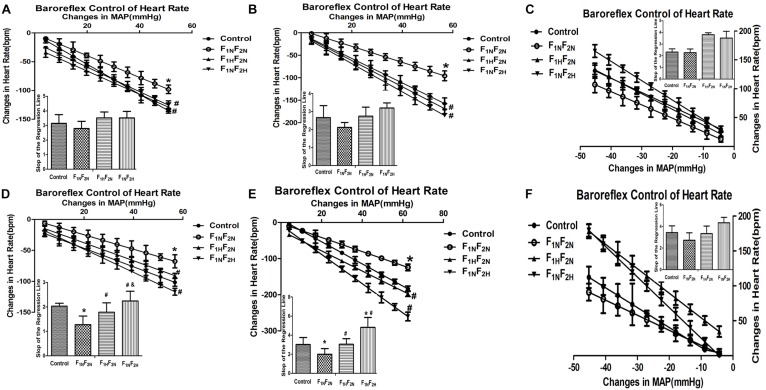
Baroreflex control of HR in conscious male offspring from Control, F_1N_F_2N_ (the offspring of RVH rats), F_1H_F_2N_ (RVH rats treated with NaHS, offspring without NaHS), F_1N_F_2H_ (maternal RVH rats treated without NaHS and the offspring with NaHS after weaning) (*n* = 6). The graph shows regression lines relating reflex bradycardia responses to Ang II and PHE or reflex tachycardia responses to SNP. Baroreflex sensitivity index expressed by the slop of the regression line. Panels **(A–C)** represent Ang II, PHE, SNP respectively at 8 weeks. Panels **(D–F)** represent Ang II, PHE, SNP respectively at 16 weeks. ^∗^*P* < 0.05 compared with Control group. ^#^*P* < 0.05 compared with F_1N_F_2N_ group. ^&^*P* < 0.05 compared with F_1H_F_2N_ group. Values are means ± SD.

The linear regression analysis of the heart rate baroreflex response to intravenous infusions of Ang II, phenylephrine (PHE), sodium nitroprusside (SNP) in male offspring are shown in Figure 3 (*n* = 6 per group). Ang II -induced baroreflex sensitivity appears blunted in the male offspring of renovascular hypertensive dams from 8 to 16 weeks. The slope of the regression line for Ang II -induced heart rate response was 1.28 ± 0.35 bpm/mmHg in the 16 weeks old male offspring of renovascular hypertensive dams which was significantly less when compared to control animal at 2.03 ± 0.11 bpm/mmHg (*P* < 0.05, Figure 3D). Additionally, compared with controls, the baroreflex sensitivity produced by PHE also exhibited a decline in the slop in F_1N_F_2N_ group (16 weeks, 3.01 ± 0.74 vs. 1.99 ± 0.58 bpm/mmHg, *P* < 0.05, Figure 3E). There was no significant difference in SNP induced baroreflex sensitivity (8 weeks, 2.32 ± 0.27 vs. 2.27 ± 0.31 bpm/mmHg, *P* > 0.05, Figure 3C; 16 weeks, 3.43 ± 0.62 vs. 2.73 ± 0.68 bpm/mmHg, *P* > 0.05, Figure 3F). The blunted effects mentioned above were reversed by treatment with H_2_S (Ang II 16 weeks, in F_1H_F_2N_ 1.78 ± 0.38 bpm/mmHg; Ang II 16 weeks, in F_1N_F_2H_ 2.24 ± 0.39 bpm/mmHg; PHE 16 weeks, in F_1H_F_2N_ and F_1N_F_2H_ were 3.04 ± 0.59 bpm/mmHg and 4.81 ± 1.04 bpm/mmHg, respectively; SNP 16 weeks, in F_1H_F_2N_ and F_1N_F_2H_ were 3.34 ± 0.7 bpm/mmHg and 4.34 ± 0.5 bpm/mmHg, respectively).

### Autonomic Nervous System Function of Male Offspring

**FIGURE 4:**
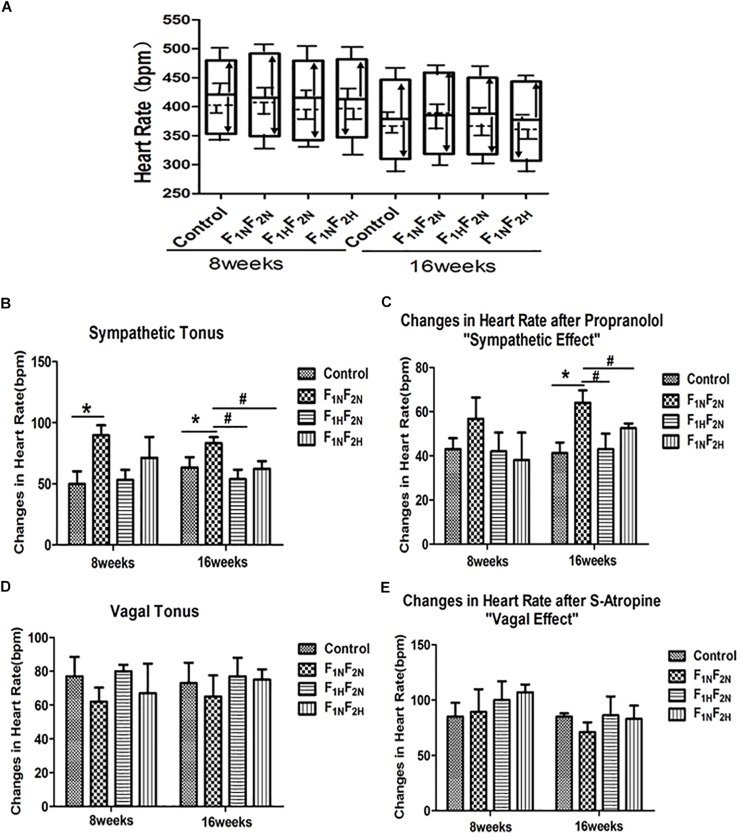
Autonomic control in male offspring of Control, F_1N_F_2N_ (the offspring of RVH rats), F_1H_F_2N_ (RVH rats treated with NaHS, offspring without NaHS), F_1N_F_2H_ (maternal RVH rats treated without NaHS and the offspring with NaHS after weaning) at 8 and 16 weeks (*n* = 6). **(A)** Bar graph representation of basal heart rate (BHR, horizontal dashed line), intrinsic heart rate (IHR, horizontal black line), sympathetic tonus (arrows pointing up), vagal tonus (arrows pointing down), heart rate following parasympathetic blockade with sulfate-atropine (upper border of the bars), heart rate responses after sympathetic blockade with propranolol (lower part of the bars). **(B)** Cardiac sympathetic tonus (bpm). **(C)** Sympathetic effect is the difference between BHR and the minimal HR obtained after propranolol alone. **(D)** Cardiac vagal tonus (bpm). **(E)** Vagal effect is the difference between maximal HR after sulfate-atropine alone and BHR.^∗^*P* < 0.05 compared with Control group. ^#^*P* < 0.05 compared with F_1N_F_2N_ group.

After autonomic blockade, the sympathetic tonus was increased in male offspring of F_1N_F_2N_ group (49.83 ± 10.52 vs. 89.4 ± 10.02 bpm *P* < 0.05) at 8 weeks. Furthermore, both the sympathetic effect (41.25 ± 4.78 vs. 64.5 ± 5.66 bpm *P* < 0.05) and sympathetic tonus (63.25 ± 8.61 vs. 83.21 ± 5 bpm *P* < 0.05) were significantly enhanced when compared to control offspring at 16 weeks. Both maternal and offspring treatment with H_2_S improved the autonomic dysfunction (Figure 4, *n* = 6 per group).

### The Plasma Level of Ang II, the Protein Expression and mRNA Level of AT1R in Offspring

**FIGURE 5:**
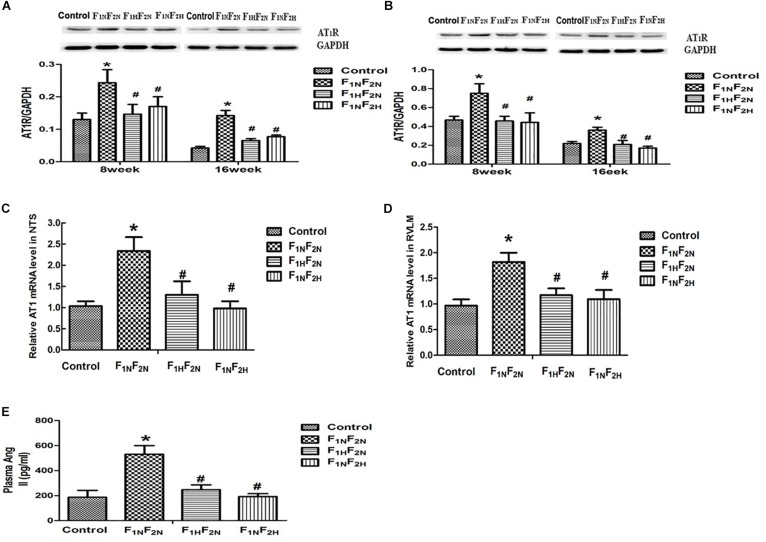
The plasma level of Ang II, and the protein and mRNA expression of AT1 in male offspring. Expression of AT1R in NTS and RVLM of offspring from Control, F_1N_F_2N_ (the offspring of RVH rats), F_1H_F_2N_ (RVH rats treated with NaHS, offspring without NaHS), F_1N_F_2H_ (maternal RVH rats treated without NaHS and the offspring with NaHS after weaning). Panels **(A,B)** representative the protein level of AT1 of male offspring at 8 weeks and 16 weeks in NTS and RVLM respectively. Panels **(C,D)** representative mRNA level of AT1 at 16 weeks. GAPDH was used to normalize. Panel **(E)** represented plasma Ang II in male offspring at 16 weeks. ^∗^*P* < 0.05 vs. Control group, ^#^*P* < 0.05 vs. F_1N_F_2N_ group. Values are means ± SD (*n* = 6–12).

Western blot analysis showed that protein level of AT1R in NTS and RVLM was elevated in F_1N_F_2N_ group compared with offspring of normotensive dams at 8 and 16 weeks. We used QPCR tested the mRNA levels of AT1R in NTS and RVLM and showed the mRNA levels of AT1R in NTS and RVLM were increased in hypertensive offspring at 16 weeks. Prenatal or postnatal treatment with H_2_S down regulated AT1R level in NTS and RVLM (Figures 5A–D). Meanwhile, the plasma level of AngII was increased in offspring of hypertensive dams at 16 weeks (Figure 5E, *n* = 6–8 per group).

### Expressions of AT1R Downstream Target Proteins in Male Offspring in NTS and RVLM

**FIGURE 6:**
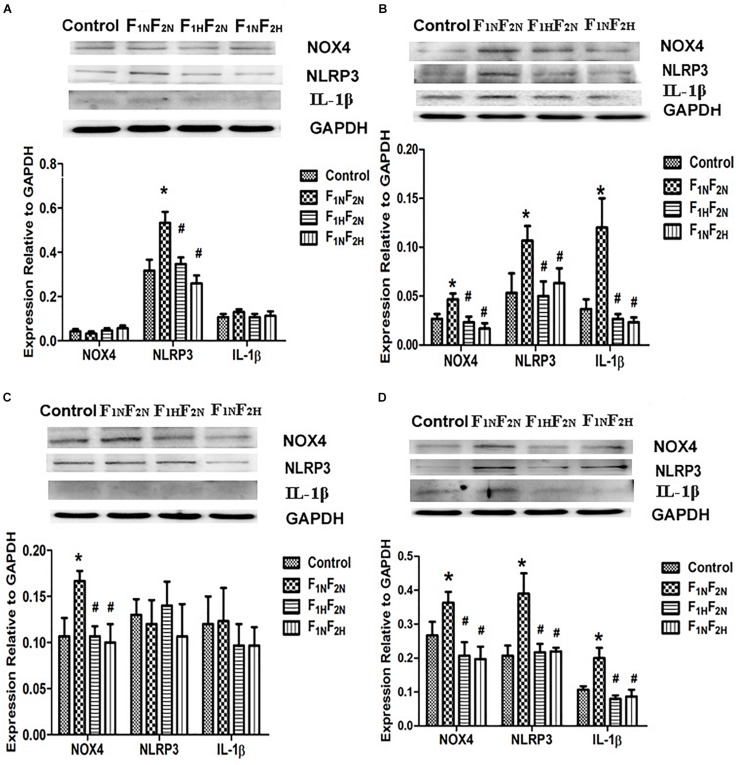
Expressions of AT1R downstream target proteins in male offspring in NTS and RVLM. Expression of NOX4, NLRP3, IL-1β in NTS and RVLM of male offspring from Control, F_1N_F_2N_ (the offspring of RVH rats), F_1H_F_2N_ (RVH rats treated with NaHS, offspring without NaHS), and F_1N_F_2H_ (maternal RVH rats treated without NaHS and the offspring with NaHS after weaning). Panels **(A,B)** representative of male offspring at 8 weeks and 16 weeks in NTS. Panels **(C,D)** representative of 8 and 16 weeks in RVLM. GAPDH was used to normalize. ^∗^*P* < 0.05 vs. Control group, ^#^*P* < 0.05 vs. F_1N_F_2N_ group. Values are means ± SD (*n* = 12).

Western blot analysis showed that protein levels of NLRP3 in NTS (Figure 6A) and NOX4 in RVLM (Figure 6C) were elevated in F_1N_F_2N_ group compared with offspring of normotensive dams at 8 weeks. Protein expression of NOX4, NLRP3 and IL-1β was increased at 16 weeks in both NTS and RVLM of F_1N_F_2N_ group, which were significantly inhibited by prenatal or postnatal treatment with H_2_S (Figures 6B,D) (*n* = 12 per group).

### Expressions of CBS in NTS and RVLM

**FIGURE 7:**
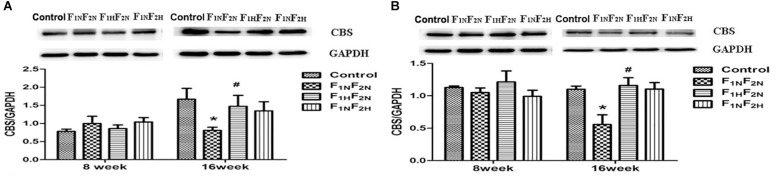
Expressions of CBS in NTS and RVLM. Panel **(A)** representative of male offspring in NTS from Control, F_1N_F_2N_ (the offspring of RVH rats), F_1H_F_2N_ (RVH rats treated with NaHS, offspring without NaHS), and F_1N_F_2N_ (maternal RVH rats treated without NaHS and the offspring with NaHS after weaning) at 8 weeks and 16 weeks. Panel **(B)** representative of male offspring in RVLM from Control, F_1N_F_2N_ (the offspring of RVH rats), F_1H_F_2N_ (RVH rats treated with NaHS, offspring without NaHS), and F_1N_F_2N_ (maternal RVH rats treated without NaHS and the offspring with NaHS after weaning) at 8 weeks and 16 weeks. GAPDH was used to normalize. ^∗^*P* < 0.05 vs. Control group, ^#^*P* < 0.05 vs. F_1N_F_2N_ group. Values are means ± SD (*n* = 12).

Protein level of CBS was decreased in male pups of F_1N_F_2N_ group at 16 weeks in NTS (Figure 7A) and RVLM (Figure 7B) (*n* = 6 per group). Either prenatal or postnatal treatment with H_2_S improved the above protein expression.

## Discussion

**FIGURE 8:**
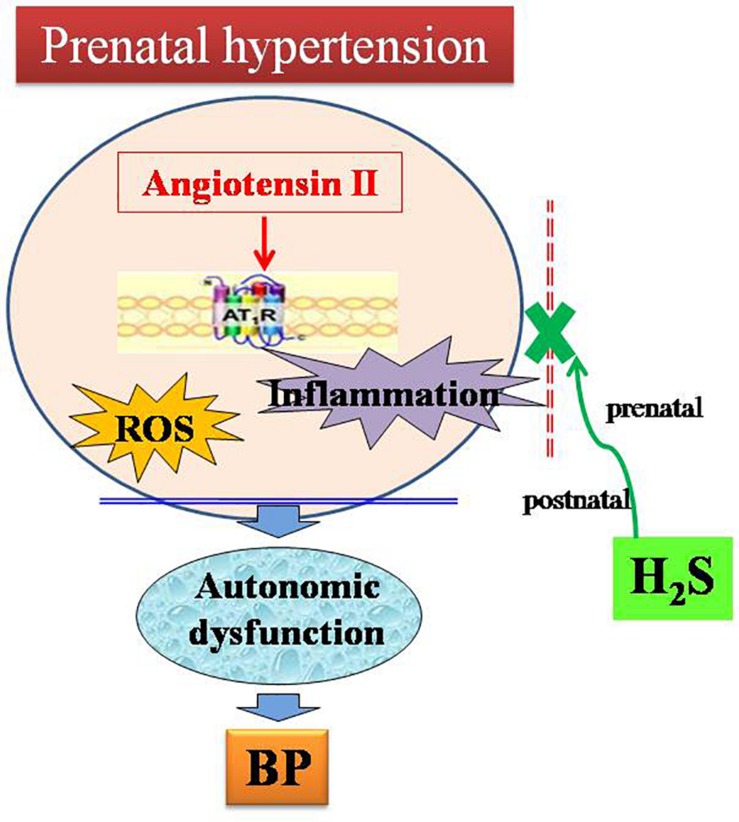
Schematic representation of the ameliorative effect of H_2_S on autonomic dysfunction in male offspring of parental hypertension.

In the present study, the main findings are as follows: (1) male offspring of renovascular hypertensive dams exhibited higher blood pressure and impaired autonomic function. (2) AT1 receptor, oxidative stress and inflammation-related protein were up regulated in male offspring of renovascular hypertensive dams. In contrast, the expression of CBS was decreased in male offspring in NTS and RVLM. (3) Either prenatal or postnatal treatment with H_2_S reduced blood pressure, improved autonomic function and inhibited activation of AT1R in hypertensive offspring. These results indicate H_2_S plays a beneficial role in improving perinatal hypertension-induced autonomic dysfunction in the offspring. H_2_S can be used for the perinatal intervention of fetal-programmed disease in the future (Figure 8).

A large number of studies have found that offspring of preeclamptic or hypertensive mothers, or of mothers with malnutrition, diabetes, low protein, high protein, and high fat diet exhibited effects on blood pressure in adulthood (Lazdam et al., 2010; Liang et al., 2013; Staley et al., 2015). However, Denton demonstrated that basal blood pressure in adult offspring of mothers with secondary 2K-1C hypertension was not increased (Denton et al., 2003). In contrast, our study showed an increased blood pressure in adult male offspring of parents with secondary 2K-1C hypertension, suggesting a paternal influence. The patrilateral factors (nutritional, lifestyle, etc) also play an important role in fetal phenotype (Li et al., 2016). Vik et al. (2014) reported that body mass index (BMI), height, blood lipids, blood pressure, and blood glucose of offspring is related with both paternal and maternal origin. In our experiment, we prepared renovascular hypertensive parents to observe the impact on blood pressure in the offspring. Compared with Denton’s study, our results suggest that paternal characteristics might play an enhanced role with maternal programming in modifications of the offspring’s phenotype.

Many basic studies and clinical trials have demonstrated that the origin and progression of hypertension are associated with dysfunction of the autonomic nervous system, especially the activation of the sympathetic nervous system (Kuchii, 1992; Ryder et al., 2016). The arterial baroreflex sensitivity is usually evaluated by reflex changes of heart rate in response to changes in arterial blood pressure, namely the arterial baroreflex control of heart rate (Ferreira-Junior et al., 2012). We performed baroreflex regulation of HR to increase in blood pressure with phenylephrine (PE) and Ang II and decrease in blood pressure with sodium nitroprusside (SNP) in conscious offspring. Both Ang II and PE induced blunted baroreflex control of heart rate in hypertensive offspring, suggesting that the blunted baroreflexes are associated with the elevated blood pressure in offspring of hypertensive rats.

In addition to the peripheral RAS, a local RAS has been demonstrated in the brain (Nakagawa and Sigmund, 2017), and angiotensin-like immunoreactivity is present in nuclei involved in blood pressure regulation (Weyhenmeyer and Phillips, 1982). Circulating Ang II can also act on the NTS or RVLM via neural projections to the area postrema, a circumventricular organs, lacking well-formed blood–brain barrier (Ferguson, 2009). It is well established that Ang II acts on the central sites involved in cardiovascular regulation to increase the sympathetic tone and reset the baroreflex, shifting the baroreflex heart rate responses to a higher pressure (Xue et al., 2003; Takahashi, 2012; Mancia and Grassi, 2014). We found that both the plasma Ang II levels and the expression of AT1R in the NTS and RVLM were increased in hypertensive offspring. These results indicate that the upregulated Ang II signals in both periphery and brain account for the blunted BRS, thereby elevating blood pressure in offspring of hypertensive dams, which is consistent with previous studies showing that Ang II receptor located on central nuclei is responsible for the blunt actions of Ang II on baroreflexes (Colover, 1985; Paton et al., 2001). Furthermore, we found that PE-induced baroreflex was also blunt in hypertensive offspring, suggesting that besides Ang II involvement of baroreflex resetting, baroreceptor responsiveness and/or controlling heart function may also be altered in hypertensive offspring. This findings warrant further study in the future.

It is also well known that Ang II exerts an inhibitory effect on the vagal tonus and a stimulatory effect on sympathetic tonus. The mechanisms involved in baroreflex modulation are related to changes in the autonomic drive to the periphery, heart and vessels (Averill and Diz, 2000; Jiang et al., 2019). In the present study, we used propranolol and atropine to block the natural sympathetic and vagal outflow to heart and showed that hypertensive offspring have an increase in sympathetic outflow without changes in the parasympathetic drive to the heart. The increase in sympathetic tonus may explain the increased baseline HR and the decreased sensitivity of the baroreflex control of HR. Interestingly however, we observed that hypertensive offspring did not present significantly alterations in cardiac vagal tonus, only a little decrease in parasympathetic tonus. The reasons for this discrepancy need to be further research.

The NTS and RVLM, two important cardiovascular centers, are involved in the regulation of BP and autonomic function (Vasquez et al., 1992; Braga et al., 2011; Sayk et al., 2015). The NLR family, pyrin domain-containing 3 (NLRP3) inflammasome is increased in several diseases (An et al., 2019; Li et al., 2019). As the most classic inflammasome, NLRP3 is critical for inflammation. Activation of the NLRP3 inflammasome promoted the secretion of inflammatory cytokines IL-1β. IL-1β is recognized as a key trigger of inflammation and is involved in sympathetic hyperactivity (Wang et al., 2019; Yin et al., 2019). Furthermore, the activation of reactive oxygen species (ROS) participates in the activation of NLRP3 inflammasome (Gross et al., 2016). It has been demonstrated that increased NADPH oxidase activity enhances ROS production, leading to hypertension. All of these enhancements including inflammation and ROS production are associated with activation of Ang II type 1 (AT1) receptor (Catt et al., 1984; Campbell, 1987; Suzuki et al., 2006; Cardinale et al., 2012; Capettini et al., 2012). In this study, we found that central AT1R was activated in both NTS and RVLM. Whereas, NLRP3 inflammasome and NOX4 (NADPH oxidase-4) were upregulated in NTS and RVLM at 8 weeks, respectively. At 16 weeks of age, the expression of AT1R, NLRP3, IL-1β, NOX4 protein in NTS and RVLM of male offspring further increased. These results implied inflammation and oxidative stress played a different role in NTS and RVLM at different time course, respectively. The synergistic interactions among Ang II, oxidative stress and inflammatory factors in these cardiovascular nuclei facilitated the development of hypertension in offspring of hypertensive dams.

Our previous study has found that the protective effect of H_2_S on artery was attributable to inhibition of vascular oxidative stress by suppressing Ang II-AT1R action (Xue et al., 2015). And H_2_S could facilitate baroreflex and inhibit sympathetic outflow (Duan et al., 2015). Similarly, maternal or offspring treatment with H_2_S also could reverse maternal hypertension-induced adverse effects mentioned in the present study. Moreover, maternal H_2_S treatment during pregnancy and lactation decreased the blood pressure of the offspring up to 4 months after birth. This long last decrease in blood pressure may be related to epigenetic modification and that mechanism of the long-lasting effect of H_2_S on blood pressure may be attributed to the reduced expression of AT1R/ROS/inflammation pathway in NTS and RVLM.

In summary, current results indicate the protective effects of H_2_S on the development of autonomic dysfunction and hypertension in offspring of hypertensive dams is associated with the down-regulation of AT1R and the inhibition of the downstream oxidative stress and inflammation in NTS and RVLM. The present study represents a new strategy for the prevention and treatment of fetal-programmed hypertension. Further investigation and clinical studies on the protective effects of H_2_S on maternal hypertensive mothers or programmed hypertensive offspring presents a promising avenue for future exploration.

## Data Availability

All datasets generated for this study are included in the manuscript and/or the supplementary files.

## Ethics Statement

The animal study was reviewed and approved by the Animal Care Committee of Hebei Medical University.

## Author Contributions

QG and YW conceived and designed the study, and prepared the manuscript. QG, XF, and HX conducted the research. XT and LX provided the essential reagents and materials. SJ and XD acquired, analyzed, and interpreted the data. All authors have approved the final version of the manuscript and agreed to be accountable for all aspects of the work.

## Conflict of Interest Statement

The authors declare that the research was conducted in the absence of any commercial or financial relationships that could be construed as a potential conflict of interest.
